# Ideal-observer models of perceptual contrast enhancement

**DOI:** 10.1186/1471-2202-14-S1-P162

**Published:** 2013-07-08

**Authors:** Jonathan Platkiewicz, Hannah Michalska, Vincent Hayward

**Affiliations:** 1 Institut des Systèmes Intelligents et de Robotique, UPMC Univ Paris 06, Paris, France; 2 Department of Electrical and Computer Engineering, McGill University, Montreal, Quebec, H3A 2A7, Canada

## 

In standard ideal-observer models of sensory cue integration [1], the perceptual estimate resulting from the combination of two cues lies in the interval bounded by the estimates of each cue separately. For example, this type of model accounts well for the psychophysical result - observers give an estimate in-between the haptic-alone and the visual-alone estimates, when asked to estimate ridges height with both vision and touch [2]. Nevertheless, a class of perceptual illusion is supposedly not accounted for by this type of model, namely contrast illusion, such as the size-weight illusion [3,4]. In the size-weight illusion, when asked to estimate the weight of two objects of the same mass but not the same size, observers estimate the larger as lighter. Using standard ideal-observer models, we showed that it is possible to account for this class of illusion provided that statistical correlation between each cue estimate is taken into account. Our argument is based on statistical inference models such as linear minimum-variance unbiased estimation, maximum a posteriori estimation, and least relative surprise estimation. This psychophysical model is general as long as the perceptual estimate deals with a physical quantity that is proportional to another physical quantity also available as a cue, such as mass and volume for a given material in the size-weight illusion.
